# Maternal Fluoride Exposure Exerts Different Toxicity Patterns in Parotid and Submandibular Glands of Offspring Rats

**DOI:** 10.3390/ijms23137217

**Published:** 2022-06-29

**Authors:** Vinicius Ruan Neves dos Santos, Maria Karolina Martins Ferreira, Leonardo Oliveira Bittencourt, Paulo Fernando Santos Mendes, Deiweson Souza-Monteiro, Karolyny Martins Balbinot, João de Jesus Viana Pinheiro, Senda Charone, Juliano Pelim Pessan, Rafael Rodrigues Lima

**Affiliations:** 1 Laboratory of Functional and Structural Biology, Institute of Biological Sciences, Federal University of Pará, Belém 66075-110, Brazil; viniciusruanpessoal@gmail.com (V.R.N.d.S.); krolmarrtins93@gmail.com (M.K.M.F.); leo.bittencourt25@gmail.com (L.O.B.); paulofsmendes@gmail.com (P.F.S.M.); deiweson.monteiro@gmail.com (D.S.-M.); sendacharone@yahoo.com.br (S.C.); 2 School of Dentistry, Institute of Health Sciences, Federal University of Pará, Belém 66075-110, Brazil; karolbalbinot@gmail.com (K.M.B.); radface@hotmail.com (J.d.J.V.P.); 3 Department of Preventive and Restorative Dentistry, School of Dentistry, São Paulo State University, Araçatuba 14801-385, Brazil; juliano.pessan@unesp.br

**Keywords:** fluoride, salivary glands, immunohistochemistry, cytoskeleton

## Abstract

There is currently a controversial and heated debate about the safety and ethical aspects of fluoride (F) used for human consumption. Thus, this study assessed the effects of prenatal and postnatal F exposure of rats on the salivary glands of their offspring. Pregnant rats were exposed to 0, 10, or 50 mg F/L from the drinking water, from the first day of gestation until offspring weaning (42 days). The offspring rats were euthanized for the collection of the parotid (PA) and submandibular (SM) glands, to assess the oxidative biochemistry and to perform morphometric and immunohistochemical analyses. F exposure was associated with a decrease in the antioxidant competence of PA in the 10 mg F/L group, contrasting with the increase observed in the 50 mg F/L group. On the other hand, the antioxidant competence of the SM glands was decreased at both concentrations. Moreover, both 10 and 50 mg F/L groups showed lower anti-α-smooth muscle actin immunostaining area in SM, while exposure to 50 mg F/L was associated with changes in gland morphometry by increasing the duct area in both glands. These findings demonstrate a greater susceptibility of the SM glands of the offspring to F at high concentration in comparison to PA, reinforcing the need to adhere to the optimum F levels recommended by the regulatory agencies. Such findings must be interpreted with caution, especially considering their translational meaning.

## 1. Introduction

Fluoride (F) exposure occurs primarily through the ingestion of F-containing water and foods, such as artificially fluoridated salt, milk, and drinking water, as well as products for topical application, including toothpastes and mouthwashes [1,2,3]. In this context, fluoridated water has been widely used as an important public health strategy to prevent dental caries, with concentrations typically ranging between 0.5 and 1.0 mg/L considered effective and safe for human consumption [3,4].

There is currently no evidence to support that the use of F at therapeutic doses is harmful to human health [5]. However, the literature shows that exposure to excessive F levels is associated with several changes in biological processes in animals, such as mitochondrial metabolism, induction of oxidative stress, interference in protein regulation, initiation of the apoptotic process and neuroinflammation [6,7]. Exposure to high concentrations of F has recently been considered a potential trigger for mitochondrial damage, causing ultrastructural damage, and modulating the respiratory chain through fission/fusion and membrane potential; the latter, in turn, can cause cell apoptosis because of increased adenosine triphosphate (ATP), reactive oxygen species (ROS) and altered Ca^2+^ influx/efflux [8].

In this sense, the interference of F in these processes can culminate in deleterious effects on the organism, promoting alterations in the central nervous in animals and skeletal systems, liver, and dental enamel in humans [9,10,11,12]. Several complications caused by exposure to excessive F levels during different stages of life have already been described in the literature [13,14,15].

Concerning the oral cavity, the chronic ingestion of above-optimum F concentrations can lead to dental fluorosis development [16]. Other structures can also be affected by exposure to F at high concentrations, such as salivary glands [17,18]. These are important organs for maintaining homeostasis in the oral environment, given that saliva (their main product) performs clearance, buffering, remineralization of dental enamel, and lubrication, in addition to aiding in speech and swallowing [19,20,21,22]. Of the numerous salivary glands of the oral cavity, about 90% of saliva production is carried out by the set of major glands, which comprise the parotid, submandibular and sublingual glands [23].

Previous studies using an adult animal model were able to associate long-term F exposure with several alterations in the parameters of oxidative biochemistry and proteomic profile pattern of the major salivary glands [17,18]. However, evidence on the effects of indirect exposure to F during gestation and lactation and on the possible repercussions on the major salivary glands of the offspring is still lacking. Thus, the objective of this study was to investigate whether maternal exposure to F at different levels during gestation and lactation could promote any biochemical and/or structural changes in the salivary glands of the offspring. The study’s null hypothesis was that the addition of fluoride to the drinking water would not promote any changes in the parameters related to the salivary glands of the offspring.

## 2. Results

### 2.1. Indirect Exposure to F during the Intrauterine and Neonatal Periods Modulated the Antioxidant Competence of Offspring Rats’ Salivary Glands

**Figure 1:**
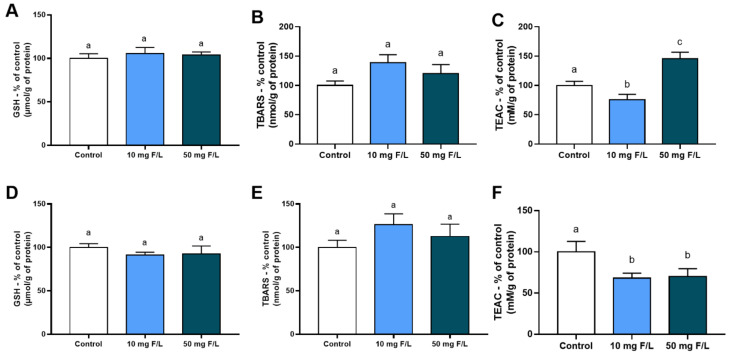
Oxidative biochemistry analyses in the parotid and submandibular glands of offspring exposed and not exposed to F, at doses of 0 mg F/L (control), 10 mg F/L, and 50 mg F/L for 42 days. In (**A**), reduced glutathione (GSH) levels in parotid glands; in (**B**) levels of thiobarbituric acid reactive substances (TBARS) in parotid glands; in (**C**), total antioxidant capacity (TEAC) of the parotid glands; in (**D**), GSH levels in submandibular glands; in (**E**) levels of TBARS in submandibular glands; in (**F**), TEAC of the submandibular glands. Quantitative results are expressed as mean ± standard error of mean, 1-way ANOVA test with Tukey’s post-test. Different letters mean statistical difference (*p* < 0.05).

Indirect exposure of the offspring to fluoridated water during the intrauterine and neonatal periods was not able to promote significant changes in reduced glutathione (GSH) levels in the parotid glands (Control: 100 ± 10.75%; 10 mg F/L: 105 ± 15.99%; 50 mg F/L: 103.74 ± 10.75%; *p* < 0.05; Figure 1A) and submandibular glands (Control: 100 ± 8.72%; 10 mg F/L: 91.15 ± 7.46%; *p* < 0.05; Figure 1D).

Likewise, the exposure of offspring to F was not able to promote significant changes in thiobarbituric acid reactive substances (TBARS) levels in the parotid glands (Control: 100 ± 15.28%; 10 mg F/L: 138.90 ± 30.21%; 50 mg F/L: 120.44 ± 34.10%; *p* < 0.05; Figure 1B) and submandibular glands (Control: 100 ± 16.42%; 10 mg F/L: 126.15 ± 28.13%; 50 mg F/L: 112.64 ± 31.55%; *p* < 0.05; Figure 1E).

However, the total antioxidant capacity (TEAC) was reduced in the 10 mg F/L group for both parotid and submandibular glands compared to the control group (Control: 100 ± 3.52%; 10 mg F/L: 75.48 ± 4.14%; 50 mg F/L: 145.5 ± 5.53%; *p* < 0.05; see Figure 1C,F). Furthermore, the TEAC was increased in the parotid of 50 mgF/L (Control: 100 ± 3.52%; 10 mgF/L: 75.48 ± 4.14%; 50 mgF/L: 145.5 ± 5.53%; *p* < 0.05) and decreased in the submandibular glands in both exposed groups in comparison to control (Control: 100 ± 6.32%; 10 mg F/L: 68.14 ± 2.72%; 50 mg F/L: 70.05 ± 4.25%; *p* < 0.05) as shown in Figure 1C–F.

### 2.2. Indirect Exposure to F Was Able to Alter the Duct Area of the Parotid and Submandibular Glands of the Offspring at Higher Levels of F, but without Changes in the Other Morphometric Parameters

**Figure 2:**
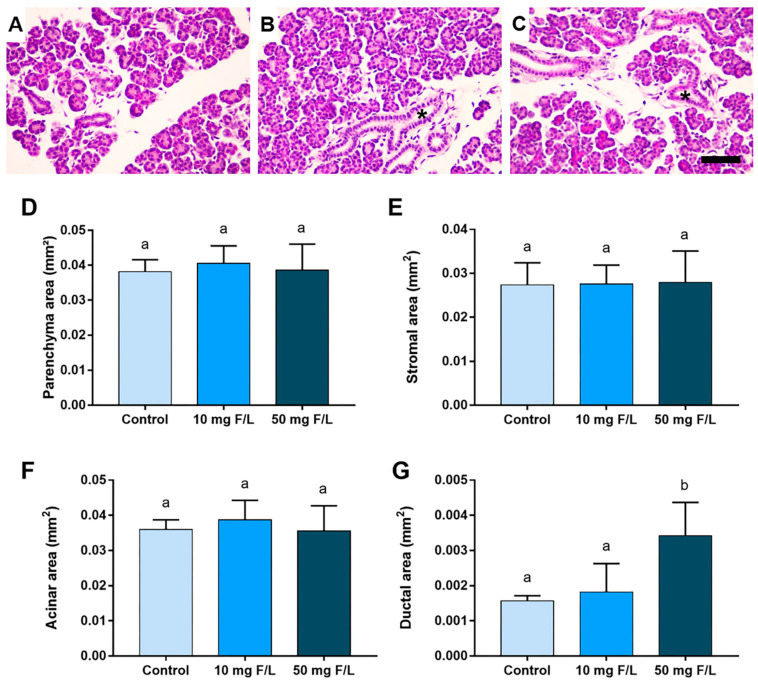
Histological image and morphometric analysis of the tissues that comprise the parotid glands of the offspring indirectly exposed to water containing 0 (control), 10, or 50 mg F/L for 42 days (*n* = 5 animals/group). (**A**–**C**) represent parotid of the 0 (control), 10 mg F/L, and 50 mg F/L groups, respectively; in (**D**), parenchyma area (mm^2^); in (**E**), stromal area (mm^2^); in (**F**), area of acini (mm^2^); in (**G**), duct area (mm^2^). Quantitative results are expressed as mean ± standard error of mean, one-way ANOVA with Tukey’s post-test. Different letters represent statistical difference (*p* < 0.05). The asterisks indicate the location of the ducts. Scale bars: 10 μm.

**Figure 3:**
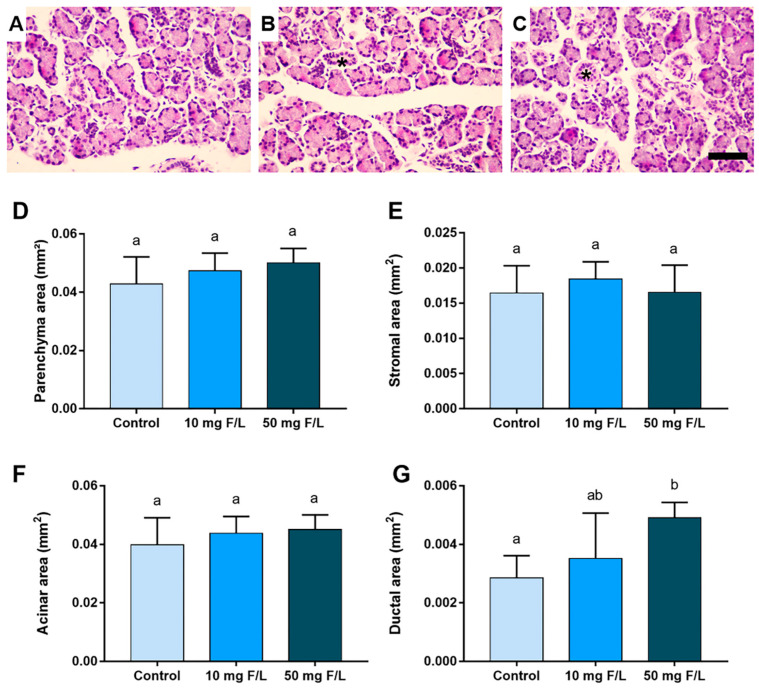
Morphometric analysis of the tissues that comprise the submandibular glands of the offspring indirectly exposed to water containing 0 (control), 10, or 50 mg F/L for 42 days (*n* = 5 animals/group). (**A**–**C**) represent the submandibular glands of the control, 10 and 50 mg F/L groups, respectively; in (**D**), parenchyma area (mm^2^); in (**E**), stromal area (mm^2^); in (**F**), area of acini (mm^2^); in (**G**), duct area (mm^2^). Quantitative results are expressed as mean ± standard error, one-way ANOVA with Tukey’s post-test. Different letters indicate statistical difference (*p* < 0.05). The asterisk indicates the location of the ducts. Scale bars: 10 μm.

The morphometric analyses detected that F at the highest concentration was able to increase the parotid gland duct area in relation to the control (0 mg F/L) and 10 mg F/L groups (*p* < 0.05) (Figure 2G). However, no significant differences were observed among the groups regarding the parenchyma area, stromal area, and acini area (Figure 2D–F, respectively), (*p* > 0.05). A similar trend was observed for the submandibular glands, in which exposure to F at 50 mgF/L increased the area of submandibular gland ducts compared to the control group (Figure 3G) (*p* < 0.05). However, no significant changes in the parenchyma, stromal, and acini areas were observed among the groups, as shown in Figure 3D–F, respectively (*p* > 0.05).

### 2.3. Indirect Exposure to F during Pregnancy and Lactation Did Not Trigger Changes in Cytokeratin-18 (CK-18) Filaments in the Salivary Glands of the Offspring

**Figure 4:**
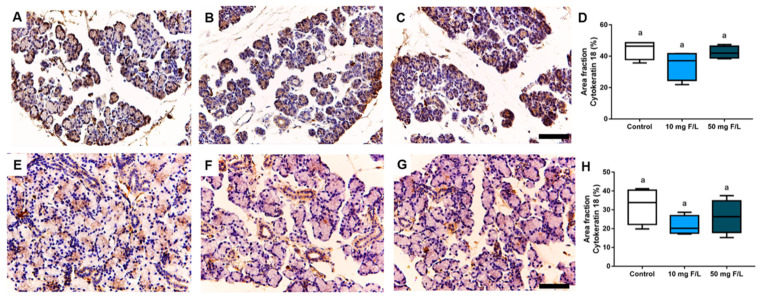
Immunohistochemical analysis of cytokeratin-18 of the tissues that comprise the parotid and submandibular glands of the offspring indirectly exposed to water containing 0 (control), 10, or 50 mg F/L for 42 days (*n* = 5 animals/group). (**A**–**C**) represent the parotid glands of the control, 10 mg F/L, and 50 mg F/L groups, respectively; in (**D**), the area fraction of the parotid gland; in (**E**), submandibular of the control group; in (**F**), submandibular in the 10 mg F/L group; in (**G**), submandibular in the 50 mg F/L group; in (**H**), the area fraction of submandibular gland. Quantitative results are expressed as mean ± standard error, one-way ANOVA with Tukey’s post-test. Different letters indicate statistical difference (*p* < 0.05). Scale bars: 10 μm.

The evaluation of samples immunostained with the anti-CK-18 showed no significant differences in the parameters of immunostained area fraction of CK-18 among the control, 10 mg F/L, and 50 mg F/L groups in the parotid and submandibular glands of the offspring rats. (Figure 4) (*p* > 0.05).

### 2.4. Indirect Exposure to F during Gestational and Lactation Periods Triggered Changes in the Immunostained Area Fraction of Myoepithelial Cells in the Submandibular Glands of Offspring Rats

**Figure 5:**
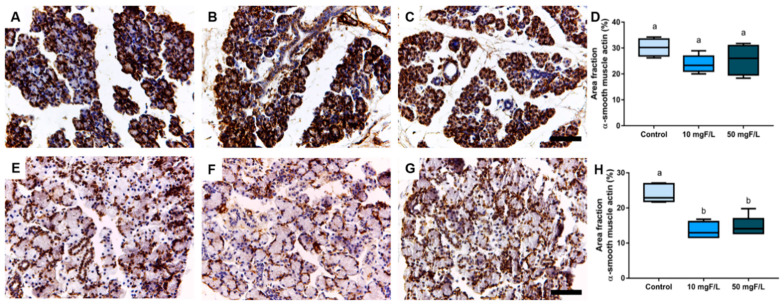
Immunohistochemical analysis of anti-α-smooth muscle actin of the tissues that comprise the parotid and submandibular glands of the offspring indirectly exposed to water containing 0 (control), 10 or 50 mg F/L for 42 days (*n* = 5 animals/group). (**A**–**C**) represent the parotid glands of the control, 10 mg F/L group, and 50 mg F/L groups, respectively; in (**D**), the area fraction of the parotid gland; in (**E**), submandibular of the control group; in (**F**), submandibular in the 10 mg F/L group; in (**G**), submandibular in the 50 mg F/L group; in (**H**), the area fraction of the submandibular gland. Quantitative results are expressed as mean ± standard error, one-way ANOVA with Tukey’s post-test. Different letters mean statistical difference (*p* < 0.05). Scale bars: 10 μm.

The evaluation of samples immunostained with the anti-α-smooth muscle actin (α-SMA) antibody showed no significant differences among groups control, 10 mg F/L and 50 mg F/L in the offspring’s parotid glands (Figure 5D) (*p* > 0.05). However, for the area fraction values obtained by analyzing the actin filaments present in myoepithelial cells of the submandibular gland, no significant difference was noted between the exposed groups (10 and 50 mg F/L; Figure 5H) (*p* < 0.05).

## 3. Discussion

This study compiled the biochemical and morphological findings of the salivary glands of rats whose mothers consumed F-containing during gestation and lactation. Our results showed an increase in the total antioxidant capacity in the parotid glands, while a decrease was observed in the submandibular glands. Such findings seem to be associated with the morphological damages observed in the submandibular glands, which showed an increase in the ductal area and a decrease in the myoepithelial cells area fraction. Taken together, the results indicate that these glands are more susceptible than the parotid gland to F-related alterations during the intrauterine and postnatal periods.

In previous studies, F exposure was shown to cause damage to the major salivary glands of adult animals [17,18]. In the present study, on the other hand, maternal exposure to F led to subtler changes than those in adult animals [17,18]. This suggests that the damage to salivary glands may be related to the form of exposure, (i.e., directly from the diet or indirectly through placenta/breastfeeding) and time of exposure, (i.e., initial vs. later stages of development), which may account for the lower susceptibility to damage in offspring compared to exposed adult organisms [24].

In fact, F levels were reported to be about 4× lower in rats indirectly exposed to F via placenta and breastfeeding in early life (10 mg F/L: 0.03 ± 0.00 μg/mL; 50 mg F/L: 0.04 ± 0.01 μg/mL) [25] compared to those in adult animals (10 mg F/L: 0.122 ± 0.00 μg/mL; 50 mg F/L: 0.142 ± 0.01 μg/mL) [26], for exposed for periods of 42 and 60 days, respectively. Interestingly, these data reveal that although lower levels are bioavailable in just weaned rats (compared with adult ones), these were able to promote significant changes in the offspring rats, such as biochemical modulations in the proteomic profile and even in the expression of brain-derived neurotrophin [25]. In addition, one must consider that the bioavailable amount of fluoride in pregnant adult rats will still be distributed to their organs, tissues, and also to the fetus.

When considering the indirect exposure protocol and blood plasma F levels in offspring, oxidative biochemical changes triggered by the concentrations used in this study could be expected, even if more subtly, since previous studies in adult animals with direct exposure have shown that these same concentrations can modulate molecular and biochemical aspects of salivary glands for a longer time [17,18]. In this regard, evidence shows that increased bioavailability of F in blood plasma may be associated with an imbalance in the redox system caused by F, which can be considered the key mechanism for these changes [27]. Therefore, GSH activity was evaluated in this study, as it is the main antioxidant defense pathway [28], in addition to malondialdehyde (MDA) dosage to assess possible oxidative damage caused by an imbalance in the redox system [29].

Concerning the TEAC levels, while an increase in oxidant activity was observed in the parotid gland, an opposite trend was verified in the submandibular gland. The literature reports the possibility of different reactions of the glands ought to biochemical modulations caused by toxic agents, since their metabolic responses may be disparate, i.e., the parotid glands present mostly aerobic metabolism, while the submandibular glands have an anaerobic metabolism [30]. Another interesting aspect is that the parotid glands have considerable levels of enzymatic antioxidants when compared to the submandibular and lingual glands [31,32]. These findings are in line with the present results since increased TEAC levels were observed in the parotid glands as a response to F exposure, possibly as a defense mechanism, in contrast to the data observed for the submandibular gland. In this scenario, the significant changes in the TEAC of the salivary glands of animals indirectly exposed to high concentrations of F, and the unchanged parameters of GSH and TEAC, indicate that the stress conditions promoted by F exposure may have occurred by pathways not investigated in the present study.

It is important to note that in addition to oxidative stress, other mechanisms, such as molecular ones, not investigated in this study, may be associated with damage by high F concentrations [33,34]. Such concentrations can trigger several alterations in the mitochondrial respiratory chain through different mitochondrial alterations, leading the organism to numerous reactions such as an increase in adenosine triphosphate (ATP), reactive oxygen species (ROS), and modulation of the dynamics of Ca^2+^ [34]. As for the area fraction parameters of the structures that compose the salivary glands, these were evaluated to allow the observation of possible morphometric changes resulting from exposure to F. Out of the parameters analyzed, only the duct area of the animals exposed to the highest concentration (50 mg F/L) was significantly increased compared to the control group. The duct system is a lining epithelium that plays an important role in modulating and conducting saliva to the oral environment [35]. This epithelium is also responsible for replacing acinar cells due to physiological or pathological processes. [36,37]. Therefore, the differences found among the study groups in this parameter could be linked to a physiological process of damage repair of the acinus caused by F overexposure, requiring further detailed studies to investigate this premise.

The histological composition of the glandular parenchyma also deserves attention. The myoepithelial cells are found at the tip of the salivary glands and are around the acinar cells that are guided by the duct systems out of the glands [35]. In this context, changes in these cells may indicate deficits in the parameters of saliva production and excretion, an important resource for maintaining homeostasis in the oral environment [19]. Functionally, these changes may be associated with xerostomia, a disease that affects the oral cavity, one of the symptoms of which is dry mouth [38,39].

Recently published studies, with other intoxicants, show that salivary glands are susceptible to damage to cell cytoarchitecture in the early stages of life [40]. The CK-18 and α-SMA are intermediate filaments that make up the cytoskeleton of acinar cells and myoepithelial cells, respectively [41,42]. The cytoskeleton, the main mechanical structure of the cell, is a dynamic network of biopolymers that includes microtubules, actin, and intermediate filaments [43,44]. Intermediate filaments can aggregate and form cytoplasmic networks responsible for cellular mechanical resistance and, in myoepithelial cells, they are also responsible for their contraction, by helping to expel saliva from the acinar cells into the duct system [45]. In the present study, the F concentrations administered were not able to promote changes in the cytoskeleton structure of acinar cells evidenced by immunohistochemistry CK-18 among the groups analyzed. When analyzing the myoepithelial cells, F was not able to cause changes in the cytoskeleton of the parotid glands in the 10 mg F/L and 50 mg F/L groups compared to the control group. However, an alteration in the submandibular glands in the 50 mg F/L group was observed, evidenced by an increase in the area fraction of α-SMA immunostaining. This difference seems to be associated with a greater susceptibility of these glands to the effects of F.

Importantly, the embryonic formation of the salivary glands occurs around the 11th embryonic day in rodents, and at gestational week 4 in humans [46]. These organs are of epithelial origin and also depend on several epithelium mesenchyme interactions until their complete formation, i.e., acini and duct system [47]. In this sense, for the formation and maintenance of the submandibular gland, it is essential that epithelium-mesenchyme interactions occur under favorable conditions so that any interference in this process can trigger modulations in these glands, making them more susceptible to damage [48].

Although the present study brings unprecedented data on the effects of indirect exposure to F on salivary glands, an important limitation deserves comment, as the period that the salivary glands were analyzed, (i.e., 21 days post-natal) might not have allowed a complete maturation of the glands. Previous studies report that between days 15–20, the myoepithelial and acinar cells already have a specific morphology, but there is still a significant reduction of organelles, which are fully present in mature cells between 30 and 40 days of postnatal life [40,49]. It is well known that salivary glands have a high renewal capacity which, associated with the maturation status of the glands, could modulate the changes found. Thus, our findings lead to new questions and encourage further studies investigating the possible long-term effects and functional repercussions.

Finally, it was noteworthy that while the use of F at 10 mg/L was not able to induce significant changes in most of the parameters analyzed, exposure at 50 mg F/L, F was shown to cause changes in oxidative biochemistry, cell structure, and the morphometry of the glands, especially in the submandibular glands. Although these data cannot be directly extrapolated to humans, the data obtained point out the safety of using F at therapeutic concentrations and reinforce the need for care regarding the overexposure to this element. Some limitations in our study must be crossed, especially regarding the possible restoration of the damages due to the epithelial turnover present in glands, which opens new possibilities for studies with different time windows. Moreover, in addition to the morphological and biochemical damages, it is necessary to investigate possible functional impairments, such as amylase activity, protein levels, and buffer capacity after intrauterine and postnatal exposure.

## 4. Materials and Methods

### 4.1. Experimental Animals

This study used pregnant Wistar rats (*Rattus norvegicus*) aged 90 days (150–200 g). Firstly, it was necessary to identify the vaginal plug, the main characteristic of the initial pregnancy period, and only after that, it was possible to start the experimental period. The rats were kept in the animal house from the Federal University of Pará (UFPA) in a light/dark cycle (lights on for 7 h) and in an acclimatized room (25 ± 2 °C). The pregnant rats were kept in individual cages with feed and water ad libitum associated with the experimental protocol. The experimental protocol was previously analyzed and approved by the Ethics Committee on the Use of Experimental Animals (CEUA), under opinion number 2718220318, and in accordance with the Guidelines for the Care and Use of Laboratory Animals [50] and Animal Research: Reporting of In Vivo Experiments (ARRIVE) [51].

### 4.2. Fluoride Exposure Protocol

Nine pregnant Wistar rats (*n* = 3/group) were randomly distributed into three experimental groups: 0 mg F/L (control group—deionized water), 10 mg F/L, and 50 mg F/L groups (after sodium fluoride solubilization in ultrapure water). All groups received water with their receptive concentrations of F and food under ad libitum conditions. Each pregnant rat generated approximately 10 offspring, comprising 30 offspring. Then, randomization was made to define 10 offspring per group. Only male animals were used in this study, and the remaining animals were used for another study. The experimental groups were conditioned to the exposure protocol with their respective concentrations for 42 days, out of which the first 21 days comprised the gestation period of the rats, and the following 21 days, the lactation period of the offspring. The doses used in this study 10 mg F/L and 50 mg F/L represent, respectively, 1–2 mg F/L and 5–10 mg F/L consumed by humans; these doses were quintupled given that the rodents’ metabolism is 5 times faster when compared to humans [25,52]. Considering these doses, a recent study by our group showed that F exposure at similar conditions as in the present study was able to promote increases in plasma F concentration (mean ± SEM) in the offspring of 0.01 ± 0.01, 0.03 ± 0.00, and 0.04 ± 0.01, respectively for water containing 0, 10 and 50 mg F/L [25].

### 4.3. Sample Preparation and Collection Procedures

At the end of the gestation and lactation period (42 days), the pups were anesthetized and euthanized intraperitoneally with ketamine hydrochloride solution (90 mg/kg) and xylazine (9 mg/kg). After the total loss of corneal reflexes, the animals intended for biochemical analysis were euthanized by cervical dislocation, and then the salivary glands (parotid and submandibular) were collected for biochemical analysis. Immediately after collection, the samples were frozen in liquid nitrogen and stored in an ultra-freezer (−80 °C) until the time of analysis. For morphometric analysis, after anesthesia, the animals were perfused with heparinized saline and 4% formaldehyde solution. Then, the glands were post-fixed in 10% formaldehyde until histological processing.

### 4.4. Biochemical Analyses

#### 4.4.1. Oxidative Stress Assessment Assays

The collected samples were washed in saline and instantly frozen with liquid nitrogen, and subsequently stored in a −80 °C freezer. To prepare for further evaluation, samples were thawed and resuspended in Tris-HCl (20 mM pH 7.4), at 4 °C by sonication (approximate concentration of 1 g/mL). The lysate was stored at −80 °C until the time of processing. From the crude homogenate, the amount of protein was determined by a method previously described [53] to normalize the lipid peroxidation results. Analyses of antioxidant and prooxidant parameters were performed to investigate changes in the biochemical balance of the parotid and submandibular glands of the offspring.

#### 4.4.2. Determination of GSH Levels

In this method, the ability of glutathione (present in the sample) in reducing 5,5-dithiobis-2-nitrobenzoic acid (DTNB; Sigma-Aldrich, St. Louis, MO, USA) to nitrobenzoic acid is tested. The determination of GSH concentrations was performed according to what was proposed by Ellman, in 1959 [54]. For this, the samples were deproteinized with 2% trichloroacetic acid and the supernatant was collected for analysis after centrifugation at 3000 rpm for 5 min. Initially, a 20 μL aliquot was taken from each sample and placed in a test tube containing 3 mL SOD buffer and 20 μL of distilled water to perform the 1st reading of the sample (T0), then 100 μL of DTNB was added and after 3 min to perform the 2nd reading of the sample (T3). The difference in absorbances (T3-T0) is proportional to the concentration of GSH (Sigma-Aldrich, St. Louis, MO, USA), expressed in μmol/g of protein, then converted to the percentage of control.

#### 4.4.3. Determination of TBARS Levels

In this analysis, the test is carried out to determine the levels of lipid peroxidation through the dosage of TBARS levels. The method to evaluate lipid peroxidation was that of Khonn and Livesedge [55] and adapted by Percário et al. [56]. After the sonication step, the gland samples were added to a solution of MDA and thiobarbituric acid (TBA) (Sigma-Aldrich, St. Louis, MO, USA) and washed in a water bath at 94 °C for 1 h. After stabilizing the temperature to room temperature, n-butyl alcohol was added, followed by vortexing and centrifugation, the supernatant was subjected to spectrophotometric reading at 535 nm to obtain the TBARS data contained in the sample, and the results were expressed in nmol/g of protein, then converted to the percentage of control.

#### 4.4.4. Determination of the TEAC Levels

The assessment of TEAC levels was tested evaluating the ability of the antioxidants present in the samples to reduce the radical 2,2′-azino-bis (3-ethylbenzothiazoline-6-sulfonic acid (ABTS). The determination of the total antioxidant capacity was performed by the TEAC technique. The samples were prepared following the method proposed by Miller et al. [57] and adapted by Re et al. [58]. The samples were added to a solution of ABTS (Sigma-Aldrich A1888, St. Louis, MO, USA) and potassium persulfate (K_2_S_2_O_8_; Sigma-Aldrich 60490). After that, the samples were analyzed by spectrophotometry read at 734 nm for 5 min. The results were expressed in mM/g of protein, then converted to the percentage of control.

### 4.5. Histological Evaluation

#### 4.5.1. Morphometric Analyses

These analyses make it possible to observe possible pathological changes in the structure of the salivary glands. For this, after perfusion, the salivary glands were removed and post-fixed in 4% formaldehyde until processing. Histological processing consisted of dehydrating the samples in increasing ethanol solutions (70%, 80%, 90%, absolute 1, absolute 2) diaphanizing in xylene, and including in histological paraffin to obtain 7 µm thick sections using a microtome manual.

For quantitative analysis, the sections obtained from the microtomy were stained with hematoxylin and eosin. For this analysis, images were taken by a color digital camera (Cyber Shot DSC W-230, 4× optical zoom, Sony, Tokyo, Japan) coupled to a microscope (1.5×, Eclipse E200, Nikon, Tokyo, Japan; in a 40× magnification) of 5 random transverse sections of the glands, with 3 fields of each section being evaluated. The direct variables of tissue morphometric evaluation, expressed in µm^2^, were: the acinar area, ductal areal, parenchyma area, and stromal area [40,59,60]. Variable values were obtained using a digital image analyzer, Image J software (NIMH, NIH, Bethesda, MD, USA)

#### 4.5.2. Immunohistochemical Analyses

These analyses allow the evaluation of essential structures of the composition of cells that structure the glandular epithelium, such as acinar, ductal, and myoepithelial cells [41,61]. The proteins investigated were CK-18 and α-SMA, which participate in salivary gland homeostasis. CK-18 is present in acinar and ductal cells and composes the intermediate filament system of these cells [62], α-SMA is a protein that indicates present in the microfilament system of the cytoskeleton in myoepithelial cells [61].

The technique used to perform immunohistochemistry was indirect immunoperoxidase [40,63]. Briefly, sections of the same samples were placed on slides treated with 3-aminopropyltriethoxysilane (Sigma-Aldrich, St. Louis, MO, USA), then deparaffinized in xylene solutions and hydrated with decreasing ethanol solutions (absolute to 70%). For antigen retrieval, they were induced with citrate buffer (pH 6.0) in a Pascal chamber (Dako^®^, Carpinteria, CA, USA) for 30 s. After this step, the sections were submerged in 3% hydrogen peroxide solution (H_2_O_2_) and methanol for 20 min to inhibition of endogenous peroxidase activity. For blocking of nonspecific sites, 1% bovine serum albumin (BSA, Sigma-Aldrich, St. Louis, MO, USA) in phosphate-buffered saline (PBS) was used for 1 h. In the next step, the slides went through an incubation process with primary anti-CK-18 (1:100, Dako^®^, Carpinteria, CA, USA) and α-SMA (1:50, Dako^®^, Carpinteria, CA, USA) antibodies. After this step, the primary antibodies were incubated with horseradish peroxidase (HRP) (Spring, Pleasanton, CA, USA) for 30 min using diaminobenzidine (DAB, Sigma-Aldrich, St. Louis, MO, USA) and the chromogens and contrasted with Mayer’s hematoxylin (Sigma^®^, St. Louis, MO, USA).

**Figure 6:**
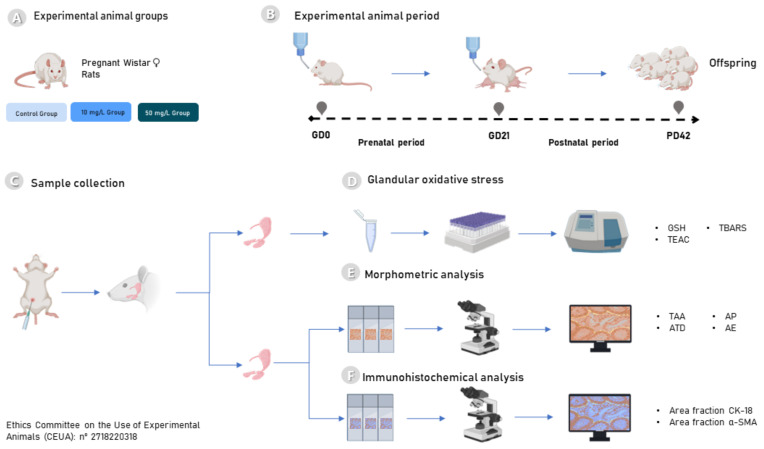
Overview of the experimental protocol. In (**A**), division of the rats into the experimental groups; in (**B**), developmental stages of the rats and their offspring before sample collection; in (**C**), collection of PA and SM glands; in (**D**), evaluation of glandular oxidative stress; in (**E**), morphometric analysis of the salivary glands and in (**F**), immunohistochemical analysis of the samples.

The area fraction (%) measurement was used for the immunostaining analysis of the immunolabeled area. Randomly, five areas were selected from each five brightfield images obtained by microscope and color camera at the same magnification as mentioned above. The areas were stained with DAB and then separated and segmented with a “deconvolution color plugin” (Gabriel Landini, http://www.dentistry.bham.ac.uk/landinig/software/software.html, accessed on 1 February 2022) and ImageJ software. Measurement was then performed on the area of the immunolabeled proteins. The results were expressed as the percentage of area immunolabeled for CK-18 or α-SMA (%). The experimental design and analyses performed are summarized in Figure 6.

### 4.6. Statistical Analysis

Data were tabulated using the GraphPad Prism 5.0 software (San Diego, CA, USA). Nonparametric data, as area fraction (%) of immunohistochemical analysis, were analyzed by Kruskal–Wallis test, followed by Dunn’s post hoc test, assuming *p* < 0.05. Parametric data, such as oxidative biochemistry assays and morphometric analysis, were submitted to one-way ANOVA, with Tukey’s post hoc test, assuming *p* < 0.05. The results of the oxidative biochemistry were expressed as a percentage of the control, with mean ± standard error, and the other results were expressed as mean ± standard error with their respective measurement units.

## 5. Conclusions

To sum up, indirect exposure to F via the placental barrier and breastfeeding was able to modulate the oxidative biochemistry of the groups exposed to F. Furthermore, F only at the highest concentration analyzed (50 mg/L) was able to trigger morphological changes in both glands investigated, as well as changes in the myoepithelial cells of the submandibular gland. Future studies are encouraged, from an environmental perspective, to better elucidate the possible molecular responses to this exposure period, and the effects of long-term exposure from the embryonic period to adulthood, since the submandibular glands have already shown damage to this period and mode of indirect exposure via progenitor-descendant.

## Data Availability

The quantitative and qualitative data used to support the findings of this study are included in the article.
